# MAGTF-Net: Dynamic Speech Emotion Recognition with Multi-Scale Graph Attention and LLD Feature Fusion

**DOI:** 10.3390/s25237378

**Published:** 2025-12-04

**Authors:** Shiyin Zhu, Yinggang Xie, Zhiliang Wang

**Affiliations:** 1 Key Laboratory of Information and Communication Systems, Ministry of Information Industry, Beijing Information Science and Technology University, Beijing 100192, China; 2023020590@bistu.edu.cn; 2 School of Computer and Communication Engineering, University of Science and Technology Beijing, Beijing 100083, China; zlw@ustb.edu.cn

**Keywords:** speech emotion recognition, feature fusion, graph neural networks, transformer encoder, multimodal learning

## Abstract

In this paper, we propose a novel speech emotion recognition model, named MAGTF-Net (Multi-scale Attention Graph Transformer Fusion Network), which addresses the challenges faced by traditional hand-crafted feature-based approaches in modeling complex emotional nuances and dynamic contextual dependencies. Although existing state-of-the-art methods have achieved improvements in recognition performance, they often fail to simultaneously capture both local acoustic features and global temporal structures, and they lack adaptability to variable-length speech utterances, thereby limiting their accuracy and robustness in recognizing complex emotional expressions. To tackle these challenges, we design a log-Mel spectrogram feature extraction branch that combines a Multi-scale Attention Graph (MAG) structure with a Transformer encoder, where the Transformer module adaptively performs dynamic modeling of speech sequences with varying lengths. In addition, a low-level descriptor (LLD) feature branch is introduced, where a multilayer perceptron (MLP) is employed for complementary feature modeling. The two feature branches are fused and subsequently classified through a fully connected layer, further enhancing the expressive capability of emotional representations. Moreover, a label-smoothing-enhanced cross-entropy loss function is adopted to improve the model’s recognition performance on difficult-to-classify emotional categories. Experiments conducted on the IEMOCAP dataset demonstrate that MAGTF-Net achieves weighted accuracy (WA) and unweighted accuracy (UA) scores of 69.15% and 70.86%, respectively, outperforming several baseline models. Further ablation studies validate the significant contributions of each module in the Mel-spectrogram branch and the LLD feature branch to the overall performance improvement. The proposed method effectively integrates local, global, and multi-source feature information, significantly enhancing the recognition of complex emotional expressions and providing new theoretical and practical insights for the field of speech emotion recognition.

## 1. Introduction

Speech emotion recognition (SER) is a critical subfield of affective computing that aims to automatically identify the emotional states of speakers through the analysis of speech signals [1,2]. As early as 1872, Darwin highlighted the existence of emotional expressions in both humans and animals in his seminal work *The Expression of the Emotions in Man and Animals* [3]. Subsequently, Ekman proposed the six basic emotions theory, which has significantly advanced the study of emotions [4].

In recent years, the rapid development of artificial intelligence and human–computer interaction technologies has propelled SER from academic research into practical applications such as intelligent customer service, virtual assistants, and mental health monitoring [5,6,7], demonstrating its substantial research potential and practical value.

Traditional SER methods primarily relied on machine learning models, such as support vector machines (SVMs), Gaussian mixture models (GMMs), and hidden Markov models (HMMs), utilizing hand-crafted acoustic features like Mel-frequency cepstral coefficients (MFCCs), spectral features, and prosodic cues. Although these methods achieved some success, they struggled with dynamic emotional expressions and exhibited limited robustness under noisy real-world conditions [8,9,10].

The advent of deep learning has significantly advanced SER. Convolutional neural networks (CNNs) and recurrent neural networks (RNNs) have enabled the automatic extraction of high-dimensional speech features, capturing long-range temporal dependencies more effectively and achieving superior recognition performance compared to traditional methods [11,12].

Despite these advancements, existing methods still face challenges in modeling complex emotional expressions. Specifically, there is a need for improved local time-frequency feature modeling, enhanced global emotional structure representation, and more efficient multi-source feature fusion strategies. Current SER approaches can be broadly categorized into three types:

- **Handcrafted Feature-Based Methods**: These methods employ manually designed acoustic features such as MFCCs and low-level descriptors (LLDs) [13], classified using traditional machine learning models [14]. While simple to implement, these features have limited expressiveness and are sensitive to noise, leading to poor generalization.
- **End-to-End Deep Learning Methods**: Deep models such as CNNs, RNNs, long short-term memory networks (LSTMs), and Transformers learn features directly from raw speech or spectrograms, improving emotion recognition by automatically extracting rich feature representations [15].
- **Multi-branch and Multi-scale Feature Fusion Methods**: Recent research emphasizes the fusion of multi-scale, multi-source features through parallel network branches to better capture complex emotional characteristics [16,17].

However, there is still considerable room for improvement in feature fusion strategies, model efficiency, and generalization capability, as recently reported in several SER studies on IEMOCAP and Emo-DB [18,19]. To address these challenges, this paper proposes a dual-branch feature fusion network for SER. The framework includes the following:

- **A Mel-spectrogram Branch**: Incorporating a Multi-scale Attention Graph (MAG) module and a Transformer encoder to effectively model both local and global emotional features;
- **An LLD Feature Branch**: Utilizing multi-layer perceptrons (MLPs) to extract and complement fine-grained frame-level emotional cues.

This study leverages a fusion network to comprehensively integrate multi-scale features for holistic emotional representation. The core objectives are to capture speech’s local emotional details, model global long-range dependencies, and efficiently fuse cross-dimensional features—enhancing SER model accuracy, generalization, and robustness to advance affective computing research and applications. Key contributions are as follows:

1. We propose a novel dual-branch architecture. One branch integrates the MAG and Transformer structures, effectively combining the advantages of multi-scale local feature extraction and global temporal modeling, thereby significantly improving the performance of speech emotion recognition tasks. The other branch deeply explores the low-level descriptor (LLD) features, further enhancing the model’s ability to capture fine-grained emotional details and improving its robustness and generalization capability.
2. We introduce a dynamic natural-length modeling mechanism that better adapts to variable-length speech utterances. To address the varying lengths of Mel-spectrogram features in natural speech, this study employs a dynamic padding and masking mechanism for batch-wise alignment and effective frame modeling.
  The padding operation ensures that the time dimensions of different samples are unified, while the mask matrix selectively masks out the padded frames during the Transformer encoding and global pooling processes, enabling feature learning and aggregation to be performed only on valid frames.This design effectively enhances the accuracy and robustness of sequence modeling.
3. Extensive experiments conducted on benchmark datasets (IEMOCAP, EMO-DB, CASIA) demonstrate the effectiveness and generalization ability of the proposed method across different languages and environments.

The remainder of this paper is organized as follows: Section 2 reviews related work. Section 3 details the proposed method. Section 4 presents experimental results and analysis. Section 5 concludes the paper and discusses future directions.

## 2. Related Work

### 2.1. Traditional Machine Learning-Based Methods

Early approaches to speech emotion recognition (SER) primarily relied on traditional machine learning models, such as Gaussian mixture models (GMMs) [20], support vector machines (SVMs) [21], and hidden Markov models (HMMs) [8]. These methods typically utilized hand-crafted acoustic features, including Mel-frequency cepstral coefficients (MFCCs), perceptual linear predictive coefficients (PLPs), and low-level descriptors (LLDs) [22].

While these traditional methods demonstrated reasonable performance in controlled environments, they exhibited several limitations. Notably, they were highly sensitive to environmental noise and variability, struggled to capture the complex and dynamic nature of emotional expressions in real-world conditions, and often suffered from poor generalization to unseen data [23]. As a result, the need for more robust and adaptive SER systems became increasingly evident.

### 2.2. Deep Learning-Based Methods

To address the limitations of traditional methods, deep learning models represented by convolutional neural networks (CNNs) and recurrent neural networks (RNNs), particularly long short-term memory (LSTM) networks, have gradually attracted increasing attention from researchers.

These models are capable of automatically learning complex features from spectrograms or raw waveforms, effectively improving the accuracy of emotion recognition [24]. CNNs are advantageous for effectively capturing local patterns in the speech spectrogram and are particularly suitable for analyzing two-dimensional features.However, CNN models generally struggle to explicitly model the sequential and contextual relationships within speech signals.

To better capture the dynamic variations in speech signals, researchers have introduced recurrent neural networks (RNNs) and their variants, such as long short-term memory (LSTM) networks and gated recurrent units (GRUs). For instance, Tzinis et al. [25] proposed an end-to-end framework that integrates multi-scale CNNs with an attention mechanism to extract multi-granularity acoustic features and fuse key frame information, thereby enhancing temporal modeling. Similarly, Latif et al. [26] highlighted the advantages of LSTM and GRU in continuous emotion recognition and complex context understanding. However, these architectures still face challenges such as vanishing gradients and limited efficiency when modeling long-range dependencies [27].

In recent years, the Transformer model, with its self-attention mechanism, has offered new opportunities for speech emotion recognition (SER). Its ability to capture long-range dependencies has been validated in both speech and emotion recognition tasks [28,29]. For instance, ref. [30] proposed a Transformer-based framework that employs multi-head self-attention to effectively capture long-range contextual information; Li et al. [31] introduced DSTCNet with a triple attention mechanism across spectro-temporal-channel dimensions, significantly improving emotional information capture; Zhao [32] proposed Dual-TBNet, which combines Transformer and BiLSTM to jointly model short- and long-term dependencies, further enhancing robustness in complex emotion recognition.

Although Transformer-based architectures exhibit strong performance in capturing long-range information, there remains room for improvement in effectively modeling multi-scale local detail features. Recent transformer-based SER models also confirm that attention over Mel-spectrograms and sample-aware training can further improve robustness [33,34].

### 2.3. Multi-Branch and Multi-Scale Feature Fusion Methods

To more comprehensively model complex emotional features, there has been a growing interest in multi-scale and multi-branch feature fusion approaches in recent years. For example, ref. [17] proposed a multi-level acoustic information fusion method based on a co-attention mechanism, which effectively improves emotion recognition performance by integrating acoustic features across different scales. In addition, graph convolutional networks (GCNs) have also been successfully applied to speech emotion modeling [35]. Chandola et al. [36] proposed SERC-GCN, which constructs a dialogue-level emotional propagation graph structure, enabling the model to achieve good performance in capturing emotional dynamics within conversations. Beyond speech-related tasks, GNNs have also been investigated from broader methodological and system perspectives. Zhao et al. proposed a flexible diffusion convolution that generalizes message passing through node-adaptive continuous diffusion processes [37]. Ma et al. reviewed key GNN acceleration strategies, including sampling, simplification, quantization, pruning, and distillation [38]. Liu et al. modeled the micro-level mobility patterns of COVID-19 cases using a deep graph diffusion framework, showing that GNNs can effectively capture complex dependencies between geometric location graphs and diffusion trajectories in real-world mobility networks [39]. Complementarily, Hang et al. proposed MEGA, a machine learning–enhanced graph-analytics framework that combines automated feature engineering with GNN-based learning to detect spambots and influential spreaders in large-scale COVID-19 Twitter graphs, highlighting the effectiveness of GNNs in relational inference and structural pattern discovery [40]. These examples underscore the generality of graph architectures and motivate our adoption of a graph-based design in the proposed MAG module.

However, such multi-scale fusion methods often involve high computational complexity and intricate model structures, and there is still substantial room for improvement in the design of effective feature fusion strategies.

In addition, to further enhance the accuracy and robustness of emotion recognition, recent research has increasingly focused on multimodal approaches that integrate speech, text, and visual information. For example, in [41], a model combining speech and text modalities was proposed, leveraging a convolutional neural network (CNN) and a pre-trained language model (BERT) to effectively improve recognition performance. Building upon this, ref. [42] introduced visual information (such as facial expression features) and proposed a multimodal model that fuses speech, text, and visual features, significantly enhancing the recognition of complex emotional states.

However, despite the performance gains, multimodal fusion methods still face several critical challenges. Notably, significant modality discrepancies often exist between features from different modalities, and the extraction of high-quality text features heavily relies on accurate automatic speech recognition (ASR) systems. In real-world environments (e.g., under low signal-to-noise ratio conditions), ASR errors can lead to degraded emotion recognition accuracy [43]. Therefore, how to achieve improved model precision while simultaneously optimizing computational efficiency and generalization performance remains one of the key research directions in the field of multimodal emotion recognition.

In summary, although significant progress has been made in speech emotion recognition, there remains considerable room for further research, particularly in simultaneously capturing both local details and global temporal features, effectively fusing different types of speech features, and ensuring model generalization capability alongside computational efficiency.

To address these issues, our work proposes a dual-branch fusion framework that separately extracts Mel-spectrogram-based and LLD-based features. By combining multi-scale local feature modeling and global sequence modeling, we aim to achieve more comprehensive emotional representation with improved efficiency and robustness.

## 3. Methods

### 3.1. Overall Framework

**Figure 1:**
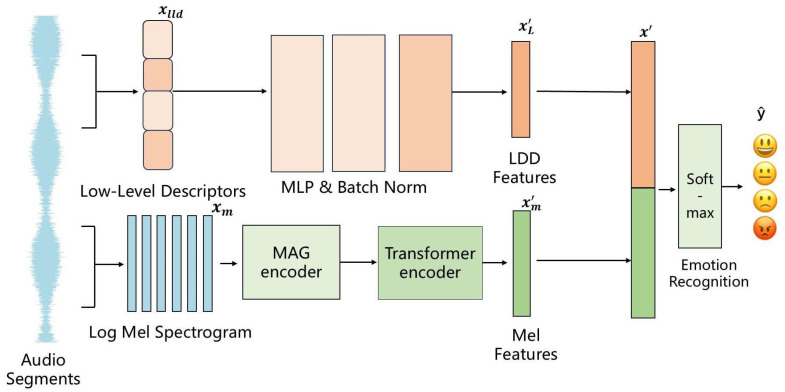
The overall framework of MAGTF-Net.

The overall architecture of the proposed MAGTF-Net is illustrated in Figure 1. The framework integrates two parallel branches to extract and fuse multi-scale and multi-source emotional features from speech signals.

Specifically, the input raw audio signals are first preprocessed to obtain two types of features:

- Log-Mel spectrogram features are extracted through short-time Fourier transform (STFT) followed by Mel-filterbank projection and logarithmic compression;
- Low-level descriptors (LLDs) are extracted using standard acoustic feature extraction techniques, including short-time energy, zero-crossing rate, spectral centroid, bandwidth, roll-off, and spectral contrast.

Formally, given an input audio sample *x*, the extracted Mel-spectrogram feature matrix $X_{m}\in R^{T\times F}$ can be expressed as

(1) $$X_{m}=\mathrm{LogMel}\left(\mathrm{STFT}\left(x\right)\right).$$

In the Mel-spectrogram branch, the input Mel-spectrogram features $X_{m}$ are first fed into the MAG encoder to perform multi-scale local feature extraction and modeling, resulting in the MAG feature representation $X_{m}^{\prime }$. Subsequently, the output features from the MAG encoder are passed into the Transformer encoder, which captures the global temporal dependencies of the speech signal and produces the Transformer output feature representation $X_{m}^{\prime \prime }$. After the Transformer processing, the extracted features combine both local and global emotional information, forming the final feature representation of the Mel-spectrogram branch.

In the LLD feature branch, the LLD feature sequence $X_{\mathrm{lld}}\in R^{T\times D}$ is extracted as

(2) $$X_{\mathrm{lld}}=\mathrm{ExtractLLD}\left(x\right),$$

where *D* represents the dimensionality of the LLD feature vector per frame. The LLD features are passed through a structure consisting of a multilayer perceptron (MLP) and batch normalization operations for feature extraction and mapping, resulting in an LLD feature representation $X_{L}^{\prime }$ that effectively captures detailed emotional information in speech. The purpose of this branch is to supplement the emotional features that may be overlooked by the Mel-spectrogram branch, thereby further enhancing the emotion recognition performance of the model.

The feature representations obtained from the two branches are fused through the feature fusion module. The fused feature representation can be expressed as

(3) $$X^{\prime }=\left[X_{m}^{\prime }\;;\;X_{L}^{\prime }\right],$$

and the concatenated feature $X^{\prime }$ is then processed by the classifier. The final emotion category prediction is obtained using the softmax function:

(4) $$\hat{y}=\mathrm{softmax}\left(W_{\mathrm{fc}}X^{\prime }+b_{\mathrm{fc}}\right),$$

where $W_{\mathrm{fc}}$ and $b_{\mathrm{fc}}$ represent the weight matrix and bias vector of the classifier, respectively.

### 3.2. Mel-Spectrogram Branch

**Figure 2:**
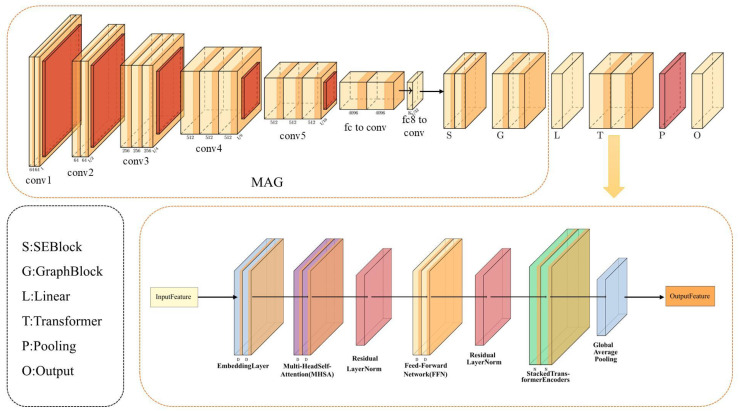
Architecture of the Mel-spectrogram feature branch.

This branch first extracts the log-Mel spectrogram features from the raw audio signals. Subsequently, multi-scale convolution and channel-attention mechanisms are applied to capture local features, followed by a local graph convolution module to enhance inter-frame dependencies. Finally, a Transformer encoder is employed to model global temporal relationships, generating a fixed-dimensional feature vector. The model structure is shown in Figure 2.

The raw audio signals are loaded using the Librosa library at a sampling rate of 16 kHz. During the data loading stage, no additional static offset removal or normalization is performed; the original waveform data is preserved for subsequent feature extraction. The STFT is first applied to convert the signal into the frequency domain, followed by a Mel filterbank projection to map the frequencies onto a perceptual scale. A logarithmic compression is then performed to obtain the log-Mel spectrogram features. The extraction parameters are set as follows: FFT size ($n_{\mathrm{fft}}$): 512; window length (win_length): 400 (corresponding to 25 ms); hop length (hop_length): 160 (corresponding to 10 ms); and number of Mel filters ($n_{\mathrm{mels}}$): 40.

Due to the varying durations of different speech samples, the extracted Mel-spectrogram features have different frame lengths *T* across samples. To enable batch-wise parallel training, a sequence alignment and masking mechanism is adopted during the data preprocessing stage. Specifically, during data loading, padding is applied to zero-pad all samples along the time dimension to match the maximum frame length within each batch. Simultaneously, a mask matrix is constructed to indicate whether each frame is valid (mask =1) or a padded frame (mask =0). The mask matrix is later utilized during the Transformer encoder and the global pooling stages to selectively model and aggregate only the valid frames, effectively avoiding the influence of padded frames on the learning process. This selective masking mechanism also reduces data dispersion introduced by variable-length utterances. Since padded positions are explicitly excluded from both attention computation and global pooling, the Transformer encoder operates on a consistent distribution of valid frames across different samples, thereby improving the stability of temporal modeling under heterogeneous speech durations.

Building upon the initially extracted log-Mel spectrogram features, a multi-scale convolution module is employed to dynamically encode local features. This module consists of five parallel one-dimensional convolutional branches, each using a different kernel size (3,5,7,9,11) to capture local variations at different temporal scales. The computation process for each branch is expressed as

(5) $$H_{i}=\sigma \left(\mathrm{Conv}1D_{k_{i}}\left(X_{m}\right)\right),\;k_{i}\in \left\{3,5,7,9,11\right\}.$$

This approach, based on varying convolutional receptive fields, enables the network to dynamically capture local temporal features without manually fixing window segmentation. Subsequently, the output of each branch is further enhanced through a channel-attention module (SE block). The SE block first performs global average pooling along the temporal dimension to generate a channel-wise descriptor:

(6) $$s=\frac{1}{T}\sum_{t=1}^{T}H_{i}\left(t\right).$$

The descriptor is then passed through two fully connected layers to generate the channel-attention weights *a*, which are multiplied with the original features along the channel dimension:

(7) $${\hat{H}}_{i}=H_{i}\cdot a.$$

Each branch output is activated by a ReLU function and then fused to obtain the dynamic local feature representation.

To further enhance the dependencies between frames within local temporal segments, a local graph convolution module (GraphBlock) is introduced after the output. Before applying the convolution-based refinement procedure described below, we explicitly construct a learnable latent acoustic graph within the GraphBlock. Unlike conventional convolution-only approaches that implicitly capture inter-channel correlations through receptive fields, the proposed design defines a graph with $c_{\mathrm{out}}$ learnable nodes along the channel dimension of the Mel-spectrogram representation. Two trainable node embedding matrices, $E_{1}\in R^{c_{\mathrm{out}}\times r}$ and $E_{2}\in R^{r\times c_{\mathrm{out}}}$ (with r=10), are used to generate the adjacency matrix:

(8) $$A=\mathrm{softmax}\left(\mathrm{ReLU}(E_{1}E_{2})\right),$$

where each row of $A\in R^{c_{\mathrm{out}}\times c_{\mathrm{out}}}$ is normalized to ensure valid outgoing edge weights. The adjacency matrix thus captures learnable correlations among latent acoustic subspaces. Graph propagation is then applied independently at each time step according to

(9) $$X_{\mathrm{graph}}\left(t\right)=X_{m}\left(t\right)A,$$

allowing each channel to aggregate information from acoustically related nodes before entering the subsequent convolutional operations.

In the GraphBlock, a two-dimensional convolution (StartConv) with a kernel size of $\left(d_{\mathrm{model}}-c_{\mathrm{out}}+1,1\right)$ is first applied to integrate local region information. Subsequently, two 1×1 convolutions followed by GELU activation functions are used to complete the nonlinear transformations:

(10) $$H^{\prime }=\mathrm{Conv}2D\left((d_{\mathrm{model}}-c_{\mathrm{out}}+1,1)\right)\left(H\right),$$

(11) $$H^{\prime \prime }=\sigma \left(\mathrm{Conv}2D\left(\left(1,1\right)\right)\left(H^{\prime }\right)\right),$$

(12) $$Z_{\mathrm{graph}}=\mathrm{Conv}2D\left(\left(1,1\right)\right)\left(H^{\prime \prime }\right).$$

Then, average pooling is performed on $Z_{\mathrm{graph}}$ along the spatial dimension, and the result is transposed back to the sequence format. A linear projection is subsequently applied to adjust the channel dimension to 128, followed by a residual connection with the original input and a layer normalization operation:

(13) $$Z_{\mathrm{local}}=\mathrm{LayerNorm}\left(Z_{\mathrm{graph}}+X_{m}\right).$$

Subsequently, $Z_{\mathrm{local}}$ is projected into a lower-dimensional embedding space through a linear layer, where the embedding dimension is set to 128. The embedded sequence is then fed into two stacked Transformer encoder layers, each consisting of a multi-head self-attention mechanism and a feedforward neural network. After residual connections and layer normalization, the global temporal feature representation $Z_{\mathrm{trans}}\in R^{T^{\prime }\times 128}$ is obtained:

(14) $$Z_{\mathrm{trans}}=Z_{\mathrm{local}}W_{\mathrm{embed}}+b_{\mathrm{embed}}.$$

The self-attention operation is defined as

(15) $$\mathrm{Attention}\left(Q,K,V\right)=\mathrm{softmax}\left(\frac{QK^{T}}{\sqrt{d_{k}}}\right)V.$$

Due to the varying sequence lengths of input samples, a weighted global average pooling is applied to the Transformer outputs using the previously constructed mask matrix. Finally, a fully connected layer is applied to map the pooled vector to the desired dimensionality, resulting in the final output of the Mel-spectrogram branch:

(16) $$z_{\mathrm{mel}}=\frac{1}{\mathrm{lengths}}\sum_{t=1}^{T}X_{t}\cdot {\mathrm{mask}}_{t},$$

(17) $$z_{\mathrm{mel},\mathrm{out}}=z_{\mathrm{mel}}W_{\mathrm{dense}}+b_{\mathrm{dense}}.$$

### 3.3. LLD Branch and Feature Fusion

**Figure 3:**
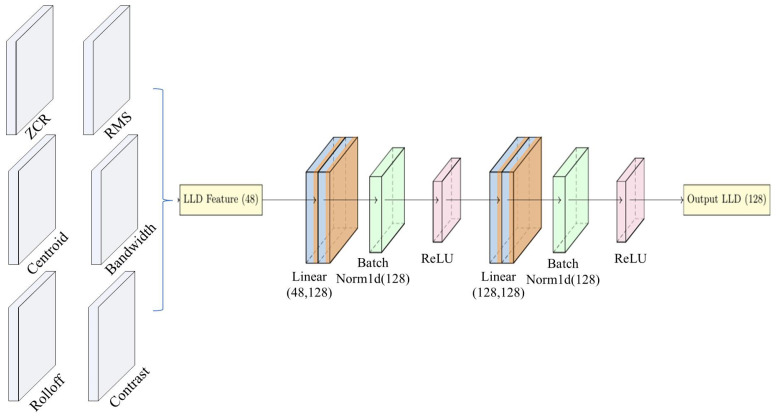
Architecture of the LLD feature branch.

To further enrich the emotional feature representation, an auxiliary feature extraction branch based on low-level descriptors (LLDs) is designed in addition to the Mel-spectrogram feature branch, as shown in Figure 3. At the final stage, the features extracted from the LLD branch are fused with those from the Mel-spectrogram branch to achieve multi-source information complementation.

The LLD features $X_{\mathrm{lld}}$ are fed into a two-layer multilayer perceptron (MLP) for feature transformation and compression. Each MLP layer consists of a linear projection followed by a ReLU activation function:

(18) $$H=\sigma \left(X_{\mathrm{lld}}W+b\right),$$

where *W* and *b* represent the weights and biases of the MLP, and σ denotes the ReLU activation function. To accommodate variable-length inputs, the LLD branch also adopts a mask-based weighted global average pooling mechanism, where only valid frames are pooled to generate a fixed-dimensional vector:

(19) $$z_{\mathrm{lld},\mathrm{out}}=\frac{1}{\mathrm{lengths}}\sum_{t=1}^{T}H_{t}\cdot {\mathrm{mask}}_{t}.$$

The feature vector output from the Mel-spectrogram branch and the feature vector output from the LLD branch are concatenated to obtain the fused feature:

(20) $$z_{\mathrm{fused}}=\left[z_{\mathrm{mel},\mathrm{out}}\;;\;z_{\mathrm{lld},\mathrm{out}}\right].$$

The fused feature is then fed into the final classifier, where it is processed through a fully connected layer followed by a softmax function to output the emotion category distribution:

(21) $$\hat{y}=\mathrm{softmax}\left(W_{\mathrm{cls}}z_{\mathrm{fused}}+b_{\mathrm{cls}}\right),$$

where $W_{\mathrm{cls}}$ and $b_{\mathrm{cls}}$ represent the weights and biases of the classifier. Through the above multi-branch feature fusion design, the model is able to integrate both local dynamic variation features and fine-grained frame-level descriptive information, thereby enhancing the robustness and classification accuracy of speech emotion recognition.

### 3.4. Interpretability of MAGTF-Net

Although MAGTF-Net adopts a deep neural architecture, several components of the model inherently support interpretability due to their structural roles in the feature extraction process. These components provide transparent links between acoustic properties and the model’s learned representations.

First, the multi-scale convolutional block introduces interpretable temporal receptive fields. Each kernel size k∈{3,5,7,9,11} captures affective information at a different temporal resolution. Small kernels emphasize short-term acoustic fluctuations such as pitch jitter, micro-prosodic variations, and abrupt spectral changes, whereas larger kernels aggregate information over broader spans to capture long-range prosodic contours. This multi-resolution design naturally decomposes emotional cues across different timescales.

Second, the graph module provides relational interpretability enhanced by graph attention mechanisms. The learnable adjacency matrix explicitly models interactions among feature channels or temporal units. On top of this structure, the graph attention mechanism assigns importance weights to these relationships, enabling the model to selectively emphasize emotionally salient spectral bands or time regions. Higher attention coefficients indicate stronger influence in the graph propagation process. This mechanism also enables future visualization techniques—such as frequency-wise heatmaps or relevance maps over time—to highlight which parts of the spectro-temporal representation contribute most to emotional classification.

Third, the Transformer encoder contributes contextual interpretability. Self-attention weights reveal how distant frames within an utterance interact. Peaks in attention often correspond to emotionally informative regions such as stressed syllables, tonal transitions, or energy changes. This complements what is learned locally by multi-scale convolution and graph attention, providing a global temporal perspective.

Finally, the LLD branch is intrinsically interpretable because its descriptors have explicit physical meaning. Features such as RMS energy, spectral centroid, bandwidth, and spectral contrast are well-established acoustic indicators of emotional expression. Their role within the fusion process therefore provides a transparent acoustic interpretation that complements the learned Mel-based representations.

Together, these components form a layered interpretability framework: multi-scale convolution explains temporal granularity, graph attention highlights salient relational patterns, Transformer attention captures contextual relevance, and LLD descriptors provide physically grounded cues. This multi-perspective interpretability enhances the transparency and credibility of MAGTF-Net.

## 4. Results

### 4.1. Datasets

To verify the effectiveness of the proposed method, experiments were conducted on three widely used speech emotion recognition datasets: EMO-DB [44], IEMOCAP [45], and CASIA [46]. These datasets cover different languages, recording conditions, and emotion categories, making them suitable for evaluating the robustness and generalization capability of the model across multilingual and multi-context scenarios.

**(1) IEMOCAP**: Released by the University of Southern California, IEMOCAP is an English multimodal emotional database consisting of dialogue utterances from 10 speakers (5 males and 5 females) across five different sessions. It includes both improvised and scripted scenarios. Each speech sample is recorded at a sampling rate of 16 kHz and annotated with emotion labels by three annotators. In this work, only the improvised utterances are selected. Following prior studies, the *excited* and *happiness* categories are merged, and four emotional categories are retained: *neutral*, *anger*, *sadness*, and *happiness*.

**(2) EMO-DB**: Released by the Technical University of Berlin, EMO-DB is a German emotional speech database comprising utterances recorded by 10 professional actors (5 males and 5 females). The dataset contains a total of 535 speech samples covering seven emotion categories, including *happiness*, *disgust*, *fear*, *boredom*, *neutral*, *sadness*, and *anger*. All samples are recorded at a sampling rate of 16 kHz.

**(3) CASIA**: Released by the Institute of Acoustics, Chinese Academy of Sciences, CASIA is a Chinese emotional speech database consisting of utterances recorded by four professional announcers (2 males and 2 females). It covers six emotional states: *anger*, *fear*, *happiness*, *neutral*, *sadness*, and *surprise*. All samples are recorded at a sampling rate of 16 kHz in a professional recording environment.

In this experiment, the total numbers of speech samples in the three datasets (CASIA, IEMOCAP, and EMO-DB) are 960, 2937, and 535, respectively. Each dataset is divided into training, validation, and test sets following an 8:1:1 ratio [31].

### 4.2. Experimental Setup

To maintain a unified experimental protocol across the three emotional speech datasets (IEMOCAP, CASIA, and EMO-DB), all datasets are divided into training, validation, and test sets following an 8:1:1 utterance-level split. This configuration is consistent with recent SER studies such as Dual-TBNet [32], and ensures comparability across multilingual corpora with different speaker and session annotations. The utterance-level splitting strategy also exposes the model to diverse speaker characteristics and acoustic conditions during training, thereby enhancing the generalization capability of the proposed framework.

During preprocessing, raw audio signals were directly used for Mel-spectrogram extraction without fixed-length segmentation. To handle variable-length Mel-spectrograms in natural speech, dynamic padding and masking were applied to align batches and model valid frames. For the LLD branch, global statistical features were computed for each entire audio sample to obtain a fixed-length feature vector, which served as the model input.

Although no global normalization is applied to the raw waveform signals, the proposed preprocessing pipeline effectively mitigates data dispersion across speakers and recording sessions. In the Mel-spectrogram branch, dynamic padding and a binary mask are adopted to align variable-length utterances within each batch. The mask ensures that only valid frames participate in the Transformer encoding and the subsequent weighted global pooling, preventing padded frames from influencing the learned feature distribution. In the LLD branch, all low-level descriptors are processed through an MLP followed by Batch Normalization, which stabilizes the feature distribution during training and reduces scale variation across multilingual datasets. Furthermore, all datasets undergo identical STFT processing, Mel-filterbank projection, and logarithmic compression, ensuring consistent feature characteristics across corpora recorded under different languages and acoustic conditions.

To ensure stable training across different datasets, the hyperparameters were tuned using an automated grid search in the early stage of experimentation. The search focused on the main training-related hyperparameters, including the learning rate, batch size, dropout ratio, and number of training epochs (with early stopping). A compact discrete grid was defined based on commonly used ranges in speech emotion recognition, where the learning rate candidates were {0.01, 0.005, 0.001}, the batch size candidates were {16, 32, 64}, and the dropout candidates were {0.1, 0.3, 0.5}. Each configuration in the grid was automatically trained and evaluated on the validation split using weighted accuracy (WA) as the primary criterion.The configuration consisting of a learning rate of 0.001, a batch size of 32, and a dropout ratio of 0.3 achieved consistently strong validation performance and was therefore adopted as the final hyperparameter setting for all experiments. The model was trained for up to 50 epochs with early stopping (patience = 25), using label-smoothing-enhanced cross-entropy loss. All model training and testing were conducted using Python 3.7 on a computer equipped with Windows 10 and an NVIDIA GeForce GTX 1050 Ti GPU.

### 4.3. Evaluation Metrics

In this study, to evaluate the performance of the proposed speech emotion recognition model, two evaluation metrics are adopted: weighted accuracy (WA) and unweighted accuracy (UA).

Weighted accuracy (WA) measures the overall classification accuracy of the model across the entire test set, defined as the ratio of correctly classified samples to the total number of samples, as shown in Equation (22). WA reflects the overall classification performance of the model and is particularly suitable for datasets with imbalanced sample distributions:

(22) $$\mathrm{WA}=\frac{TP+TN}{TP+TN+FP+FN}.$$

Unweighted accuracy (UA) is calculated by first computing the recall for each emotion category and then averaging the recall values across all categories, as defined in Equation (23). Recall is defined as the ratio of correctly predicted samples to the actual number of samples in each category. UA effectively evaluates the model’s balanced classification ability across different emotion categories and mitigates the bias that may occur in datasets with imbalanced class distributions:

(23) $$\mathrm{UA}=\frac{1}{C}\sum_{i=1}^{C}\frac{TP_{i}}{TP_{i}+FN_{i}}.$$

### 4.4. Comparative Analysis

To further demonstrate the advantages of each component in the proposed Mel-spectrogram branch model, a series of comparative experiments were conducted. The baseline model selected for comparison is CNN-BLSTM [47], which has been widely validated as a robust and efficient architecture in the field of speech processing. The CNN component can effectively capture local resonance patterns and spectral variation information at a lower level, while the BLSTM component comprehensively models emotional trends, intonation fluctuations, and temporal dependencies at a higher level within speech segments.

The experimental setup is as follows:

(a) A clean CNN-BLSTM baseline model was trained and evaluated under the local experimental environment and parameter settings to analyze its emotion recognition performance;
(b) The baseline CNN-BLSTM model was modified by replacing the fixed-length slicing of the audio source with the dynamic sequence modeling approach proposed in this study;
(c) Based on the dynamic sequence modeling version of CNN-BLSTM, the CNN module was replaced with the proposed MAG module, forming the MAG-BLSTM model;
(d) Based on the dynamic sequence modeling CNN-BLSTM model, the BLSTM module was replaced with the Transformer encoder, resulting in the CNN-Transformer model.

All the above experiments were conducted on the IEMOCAP and EMO-DB datasets, and their performances were compared with that of the final model proposed in this paper. The experimental results are summarized in Table 1 and Table 2. In the tables, models with the prefix “D” indicate that the dynamic sequence modeling method was adopted.

From the above experimental results (a–b), it can be observed that compared to the baseline CNN-BLSTM model, the dynamic sequence modeling method proposed in this study significantly improves emotion recognition performance. On the IEMOCAP dataset, WA increased from 53.64% to 62.79%, and UA improved from 61.04% to 63.82%. On the EMO-DB dataset, WA improved from 68.52% to 70.37%, and UA increased from 63.71% to 70.62%.

From the experimental results (b–d), it can be further observed that each module of the proposed model provides performance gains compared to the traditional CNN-BLSTM model. After replacing the CNN module with the proposed MAG module, WA on IEMOCAP improved from 62.79% to 69.83%, and UA from 63.82% to 67.57%. On EMO-DB, WA improved from 70.37% to 75.93%, and UA from 70.62% to 76.56%. Replacing BLSTM with the Transformer encoder further enhanced performance. Finally, the proposed MAG-Transformer achieved the best WA and UA, verifying the rationality and superiority of the model design.

### 4.5. Performance Analysis

To analyze the performance of our model on different emotions across the three datasets which is shown in Table 3, we conducted a statistical analysis of various emotion recognition metrics and plotted the corresponding confusion matrices.

**Figure 4:**
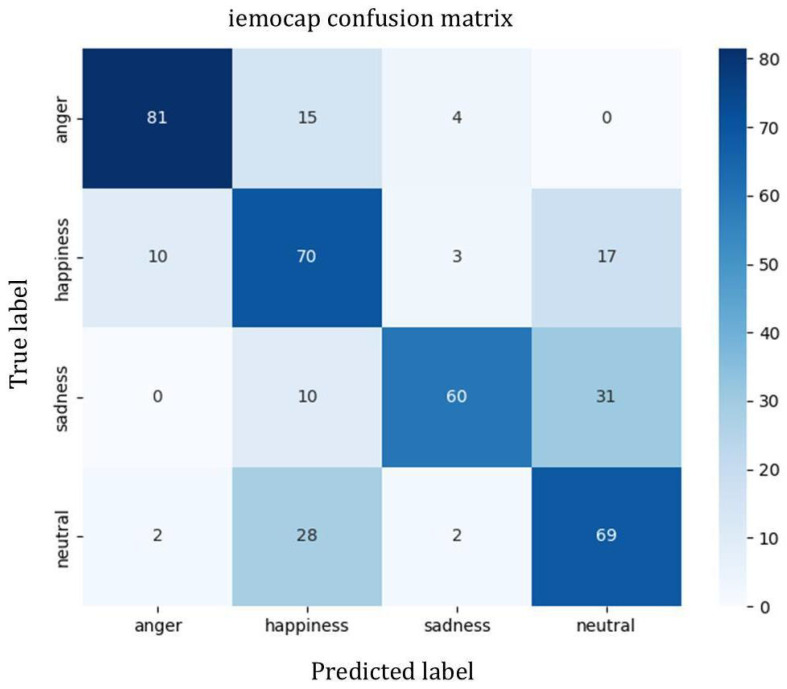
Confusion matrix of the model on the IEMOCAP dataset.

**Figure 5:**
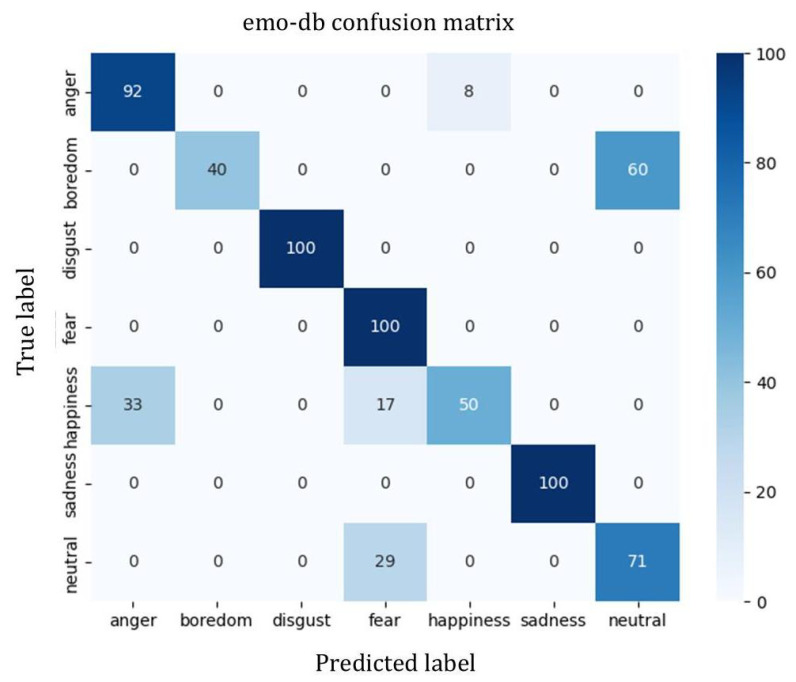
Confusion matrix of the model on the EMO-DB dataset.

**Figure 6:**
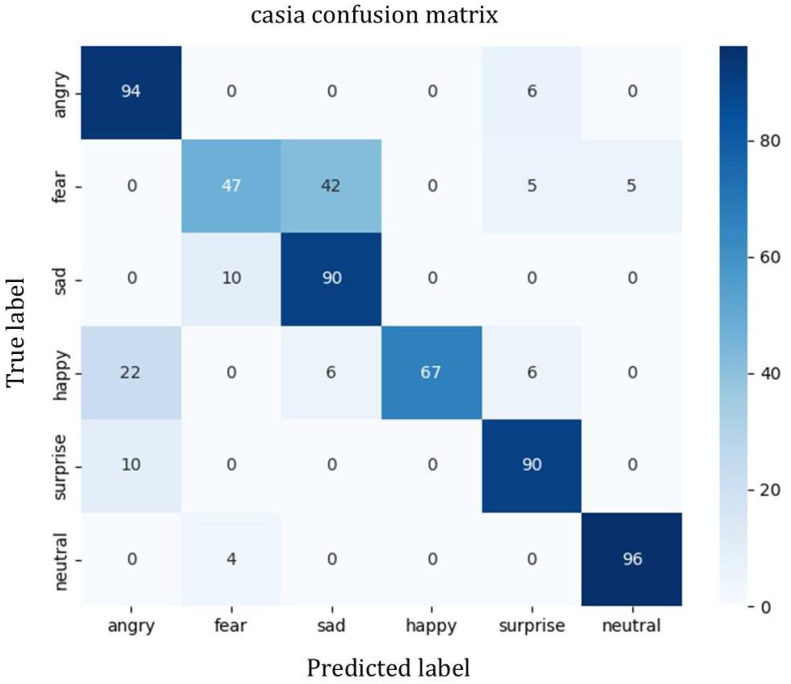
Confusion matrix of the model on the CASIA dataset.

Based on Table 3 and Figure 4, Figure 5 and Figure 6, it can be observed that on the CASIA dataset, the model achieves recognition accuracies as high as 98% for happiness, neutral, and sadness emotions, demonstrating overall stable performance. However, there is some confusion between anger and surprise, mainly due to the similarity in emotional polarity between these two categories.

On the IEMOCAP dataset, neutral emotion achieves the best recognition performance, while there are some misclassifications between anger and happiness, indicating that the model still has room for improvement in distinguishing high-arousal emotions under natural English conversational settings.

For the EMO-DB dataset, despite its smaller sample size, the model still achieves high recognition rates across most emotional categories, demonstrating good generalization capability. Overall, the model exhibits good stability and adaptability across the three datasets, showing particular strength in recognizing neutral and low-arousal emotions, and also performing well on small-sample datasets such as EMO-DB and CASIA.

We also collected recent studies in the field of speech emotion recognition for comparative analysis. As shown in Table 4, the recognition results are categorized based on different datasets.

It can be observed that the proposed MAGTF-Net achieves superior recognition performance on the IEMOCAP dataset compared with previous approaches. EMO-DB is a German speech emotion dataset and CASIA is a Chinese emotional dataset; both are relatively small in size. Our model maintains competitive performance across these multilingual datasets, confirming its robustness and generalization capability.

Finally, to further analyze the contributions of each component, ablation experiments were conducted on the EMO-DB dataset. (1) To verify the importance of the LLD feature branch, the branch was removed to evaluate its complementary effect. (2) To evaluate the graph-based local modeling, the GraphBlock layer within the MAG module was also removed for comparison.

As shown in Table 5, removing the LLD branch (-LLD) resulted in a decrease of 18.4% in UA and 14.3% in WA, indicating that low-level acoustic features play an important role in modeling emotional information. Similarly, removing the GraphBlock module (-GraphBlock) led to a 9.3% decrease in UA and a 6.9% decrease in WA, demonstrating that the graph structure positively contributes to capturing the internal relationships within Mel features.

In addition, to further validate the effectiveness of the proposed multi-scale design, we performed an extended kernel-size ablation study on the EMO-DB corpus using both single-kernel and reduced multi-kernel configurations. As shown in Table 6, certain single-scale settings—for example, k=5 and k=11—achieve relatively high UA scores (80.58% and 80.23%), reflecting the fact that EMO-DB is a highly controlled studio-recorded dataset in which emotional cues are dominated by a small number of characteristic temporal scales.

However, the full multi-scale configuration {3,5,7,9,11} obtains the highest weighted accuracy (WA = 83.33%) among all tested variants, indicating that aggregating multiple temporal receptive fields provides more stable and balanced modeling of emotional expressions. Since WA is the most reliable metric under class-imbalance conditions, this result demonstrates that the full MAG configuration remains the most robust design on EMO-DB. Compared with reduced multi-scale subsets such as {5,7,9} or {7,9,11}, the full configuration preserves complementary short-, mid-, and long-range acoustic dynamics, thereby offering stronger generalization capability.

These results confirm that although individual temporal scales may achieve strong performance on certain controlled datasets, combining multiple temporal resolutions ensures stable behavior across diverse affective speech conditions.

## 5. Discussion and Conclusions

The proposed MAGTF-Net integrates multi-scale acoustic analysis, graph-based relational modeling, and complementary handcrafted descriptors to provide a unified framework for robust speech emotion recognition. By jointly capturing fine-grained local fluctuations and broader temporal–prosodic structures, MAGTF-Net achieves competitive or superior performance across English (IEMOCAP), German (EMO-DB), and Chinese (CASIA) emotional speech datasets. The fusion of Mel-based dynamic features and LLD-based global descriptors further enhances generalization under diverse speakers and recording conditions.

Despite these encouraging results, variations in recognition performance across emotional categories indicate that cross-linguistic and cross-cultural factors remain an intrinsic challenge. English and German primarily express affective cues through stress, intensity, and rhythm, whereas Mandarin Chinese encodes lexical meaning through pitch contours, which may overlap with or obscure emotional intonation. These differences partly explain why emotions such as happiness, surprise, or confusion exhibit inconsistent acoustic manifestations across languages. Future research into domain adaptation and cross-cultural representation learning may help mitigate such disparities.

Although the structural components of MAGTF-Net, such as multi-scale convolution, graph attention, and handcrafted LLD descriptors, provide inherent interpretability, post hoc explainability techniques also play an important role in analyzing model behavior. Applying methods such as SHAP or LIME to MAGTF-Net, however, is non-trivial because the model operates on variable-length acoustic sequences, incorporates dynamic padding and masking, and contains multiple interacting branches. These characteristics violate the fixed-dimensional input assumptions underlying most post hoc interpretability frameworks. Extending SHAP-based or attribution-based techniques to sequence-level emotional modeling therefore represents a promising direction for future work and may further enhance model transparency.

From a deployment perspective, the combination of multi-scale convolution, graph reasoning, and Transformer encoding introduces non-trivial computational overhead, which may limit real-time applicability in conversational assistants or embedded affective systems. While this study focuses on offline evaluation, exploring lightweight variants—such as reducing kernel-scale redundancy, compressing graph embeddings via low-rank or sparse formulations, pruning or narrowing the Transformer encoder, or adopting knowledge distillation—represents a promising direction for improving efficiency without sacrificing performance.

Overall, MAGTF-Net offers a flexible foundation for modeling emotional cues across languages and recording conditions. Continued progress on cross-cultural generalization, explainability extensions, and lightweight model design will further extend the applicability of the framework and deepen the scientific understanding of emotional expression in diverse linguistic and computational environments.

## Figures and Tables

**Table 1:** Performance comparison on the IEMOCAP dataset.

Models	WA (%)	UA (%)
(a) CNN-BLSTM	53.64	61.04
(b) D_CNN-BLSTM	62.79	63.82
(c) D_MAG-BLSTM	69.83	67.57
(d) D_CNN-Transformer	66.38	64.56
(e) MAG-Transformer	69.15	70.86

**Table 2:** Performance comparison on the EMO-DB dataset.

Models	WA (%)	UA (%)
(a) CNN-BLSTM	68.52	63.71
(b) D_CNN-BLSTM	70.37	70.62
(c) D_MAG-BLSTM	75.93	76.56
(d) D_CNN-Transformer	77.78	74.71
(e) MAG-Transformer	83.33	79.11

**Table 3:** Detailed Performance of Our Model on Three Datasets.

	IEMOCAP			CASIA			EMO-DB		
	P (%)	R (%)	F1 (%)	P (%)	R (%)	F1 (%)	P (%)	R (%)	F1 (%)
Angry	66.67	81.48	73.33	71.43	93.75	81.08	85.71	92.31	88.89
Sad	83.78	59.62	69.66	67.86	90.48	77.55	100.00	100.00	100.00
Neutral	72.65	68.55	70.54	96.15	96.15	96.15	62.50	71.43	66.67
Happy	59.26	69.57	64.00	100.00	66.67	80.00	75.00	50.00	60.00
Disgust	–	–	–	–	–	–	100.00	100.00	100.00
Fear	–	–	–	75.00	47.37	58.06	76.92	100.00	86.96
Surprise	–	–	–	85.71	90.00	87.80	–	–	–
Bored	–	–	–	–	–	–	100.00	40.00	57.14

**Table 4:** Comparative results of speech emotion recognition on different datasets.

Models	CASIA	IEMOCAP	EMO-DB
DNN-ELM [48]	–	0.580	0.809
3DRNN+Attention [49]	–	0.647	0.828
Dual-TBNet [32]	0.957	0.648	0.841
DenseNet-GRU [50]	0.800	0.631	0.820
SVM+Decision Tree [51]	0.853	–	0.858
CNN+ChannelAttention [52]	0.887	–	0.846
Wav2Vec2.0 [53]	–	0.631	0.820
Ours	0.812	0.700	0.812

**Table 5:** Ablation study results on the EMO-DB dataset.

Models	UA	WA
-LLD	0.649	0.648
-GraphBlock	0.740	0.722
FusionNet	0.833	0.791

**Table 6:** Kernel-size ablation study on EMO-DB.

Kernel Set	UA (%)	WA (%)
{3}	70.81	66.67
{5}	80.58	79.63
{7}	71.37	68.52
{9}	66.02	61.11
{11}	80.23	77.78
{3,5,7}	66.16	68.52
{5,7,9}	77.52	75.93
{7,9,11}	80.27	78.33
{3,5,7,9,11} (Full-MAG)	79.11	83.33

## Data Availability

The datasets used in this study are publicly available at: IEMOCAP—https://sail.usc.edu/iemocap/ (accessed on 12 October 2025), EMO-DB—https://emodb.bilderbar.info/ (accessed on 12 October 2025), and CASIA—https://link.gitcode.com/i/b1a764ad5209d4d6a08667524ef00c43?uuid_tt_dd=10_19016666450-1746733703175-714152&isLogin=9&from_id=143074127 (accessed on 12 October 2025).
